# Effectiveness of Biphasic Calcium Sulfate Cement for the Prevention of Local Bleeding After Oral Surgery in Patients on Anticoagulants

**DOI:** 10.7759/cureus.106272

**Published:** 2026-04-01

**Authors:** Damian Dudek, Maciej Jagielak, Edyta Reichmann-Warmusz, Gregori M Kurtzman

**Affiliations:** 1 Dentistry, Medical University of Silesia, Katowice, POL; 2 Oral and Maxillofacial Surgery, Orthognathic and Maxillofacial Surgery Clinic, Raszyn, POL; 3 Histopathology, School of Medicine With the Division of Dentistry, Medical University of Silesia, Katowice, POL; 4 General Dentistry and Implantologist, Private Practice, Maryland, USA

**Keywords:** 3d bond, anticoagulants, biphasic calcium sulfate, extraction hemostasis, oral surgery, socket grafting

## Abstract

Introduction and aim: Anticoagulant therapy with dihydroxycoumarin derivatives (DD) is often related to local bleeding during oral surgery. The purpose of this study was to determine whether 3D Bond® (Augma Biomaterials, Katzir, Israel), a biphasic calcium sulfate regenerative bone cement, allows for the management of bleeding during and after oral surgery without discontinuation of anticoagulant therapy. The aim of the study was to investigate whether 3D Bond® biphasic calcium sulfate cement is effective in supporting local hemostasis and wound healing in patients using chronic anticoagulant therapy (dihydroxycoumarin derivatives, DD).

Methods: The study was divided into a control group and a study group. Disorders of plasma coagulation parameters were confirmed in both groups one day prior to surgery by a standard international normalized ratio (INR) test. The control group consisted of 20 patients with cardiovascular diagnoses who were discontinued on their anticoagulation therapy before extractions of erupted teeth, impacted teeth, and roots. In the study group, 20 patients with various cardiological disorders underwent extractions of erupted teeth, impacted teeth, and roots without anticoagulant treatment discontinuation. 3D Bond® was used topically with a single suture and applied compression in the study group. In the control group, standard collagen sponge compression was utilized. Sutures were removed seven to 10 days after tooth extraction. The visual assessment of bleeding was performed five times within 10 days after surgery. Wound healing and pain following surgery for 10 days were also evaluated in both groups.

Results: Clinical evaluation of the study group revealed no local bleeding in 19 patients (95%) during follow-up. Local bleeding was observed in one patient (5%) at day three; however, it was residual. In the control group, no local bleeding was noted in 16 patients (80%) during follow-up. Local bleeding was observed in four patients (20%) in the control group at day three. All wounds were properly healed, except for the four patients with local bleeding (20%) in the control group.

Conclusions: 3D Bond®, which consists of 100% biphasic calcium sulfate bone cement, is effective in inhibiting localized bleeding following oral surgery without requiring discontinuation of dihydroxycoumarin derivative medication. In addition, the clinical effectiveness of 3D Bond® in socket preservation of post-extraction alveolar sockets was observed and is comparable with other biphasic calcium sulfate cements.

Clinical relevance: Many patients are prescribed anticoagulants by their physician as part of their ongoing health management. This often leads to bleeding during and following dental surgery, such as extractions, soft-tissue procedures, and implant-related treatment. Discontinuing or decreasing the patient's anticoagulants poses potential medical risks. Utilization of 3D Bond®, a biphasic calcium sulfate regenerative bone cement, allows management of bleeding during and after those dental procedures that may cause bleeding while continuing the patient on the prescribed anticoagulants.

## Introduction

Oral anticoagulant therapy remains a cornerstone in the prevention of thromboembolic events in patients with atrial fibrillation, prosthetic heart valves, venous thromboembolism, and ischemic heart disease. It is estimated that between 1% and 2% of the adult population in developed countries is maintained on long-term vitamin K antagonist (VKA) therapy, most commonly warfarin or acenocoumarol [1-3]. With an aging population and increasing cardiovascular comorbidity, dental practitioners are encountering a growing number of patients requiring invasive oral surgical procedures while receiving chronic anticoagulation.

Vitamin K antagonists exert their effect by inhibiting hepatic synthesis of clotting factors II, VII, IX, and X, thereby prolonging prothrombin time and increasing the international normalized ratio (INR) [1,3]. Although therapeutic anticoagulation reduces the risk of systemic thromboembolism, it is associated with an increased incidence of perioperative bleeding. Reported rates of post-extraction bleeding in anticoagulated patients vary widely in the literature, ranging from approximately 2% to 13%, depending on INR levels, number of teeth removed, and local hemostatic measures utilized [4-6].

Historically, interruption of anticoagulant therapy or bridging with low-molecular-weight heparin (LMWH) was recommended prior to invasive dental procedures. However, accumulating evidence demonstrates that temporary discontinuation of VKAs may expose patients to significant thromboembolic risk without substantially reducing postoperative bleeding complications [6-8]. Contemporary guidelines therefore support continuation of therapeutic anticoagulation for most minor oral surgical procedures when effective local hemostatic techniques are employed [2,7,9].

Numerous local hemostatic strategies have been investigated to mitigate bleeding risk in anticoagulated patients, including suturing, collagen sponges, oxidized cellulose, fibrin adhesives, tranexamic acid mouth rinses, and topical thrombin preparations [9-13]. These modalities primarily function through mechanical tamponade or enhancement of clot stabilization. While generally effective, bleeding episodes still occur, particularly in cases involving multiple extractions, inflamed tissues, or elevated INR values.

Biphasic calcium sulfate-based materials have traditionally been utilized as regenerative grafting agents due to their biocompatibility, resorbability, and ability to harden in situ. Beyond their regenerative applications, calcium-containing materials may theoretically enhance local coagulation through calcium ion release, a key cofactor in multiple stages of the coagulation cascade. The in-situ hardening property may also provide mechanical sealing of cancellous bone and extraction socket microvasculature, potentially improving local hemostatic predictability.

3D Bond® (Augma Biomaterials, Katzir, Israel) is a biphasic calcium sulfate regenerative cement designed for socket preservation and ridge maintenance. Its handling properties allow placement directly into extraction sites, where it sets into a stable scaffold. While its regenerative capacity has been clinically documented, limited data exist regarding its adjunctive role in hemostasis among patients maintained on chronic anticoagulant therapy. Biphasic calcium sulfate is a hard-setting material that clogs the defect with its blood flow via capillaries, stopping bleeding. This closes off those microvessels controlling site bleeding. Additionally, the calcium ions in the 3D Bond can potentially support the coagulation cascade, which activates as a cofactor for prothrombin transition to thrombin and stabilizes the clot.

Given the increasing clinical need to safely manage anticoagulated patients in outpatient dental settings, further investigation into materials that may provide both hemostatic control and regenerative benefit is warranted. Accordingly, the aim of this study was to evaluate whether 3D Bond® biphasic calcium sulfate cement supports effective local hemostasis and wound healing in patients receiving chronic vitamin K antagonist therapy without interruption of anticoagulation.

## Materials and methods

Twenty consecutive patients who were referred for planned oral surgery were enrolled. All patients (nine women and 11 men) were on an oral anticoagulant with vitamin K antagonists due to coronary artery bypass graft surgery (CABG), surgical implantation of artificial valves, pulmonary embolism in an interview, or chronic atrial fibrillation. The exclusion criteria included a lack of agreement for using the 3D Bond® graft. Data from blood tests at admission, including international normalized ratio (INR) levels, were obtained from the patients. Informed consent was obtained from each patient in the study prior to initiation of treatment.

The control group is a group of 20 patients (11 women and nine men) who were discontinued from anticoagulation therapy in the year 2005-2006. A bridging therapy was utilized with subcutaneous administration of low-molecular-weight heparin (LMWH, enoxaparin 40-80 mg) until the INR fell below or equal to 1.5. To monitor the daily heparin dose, INR ratio, prothrombin time indicator, and activated partial thromboplastin time (APTT) were monitored, all within the normal accepted range.

**Figure 1:**
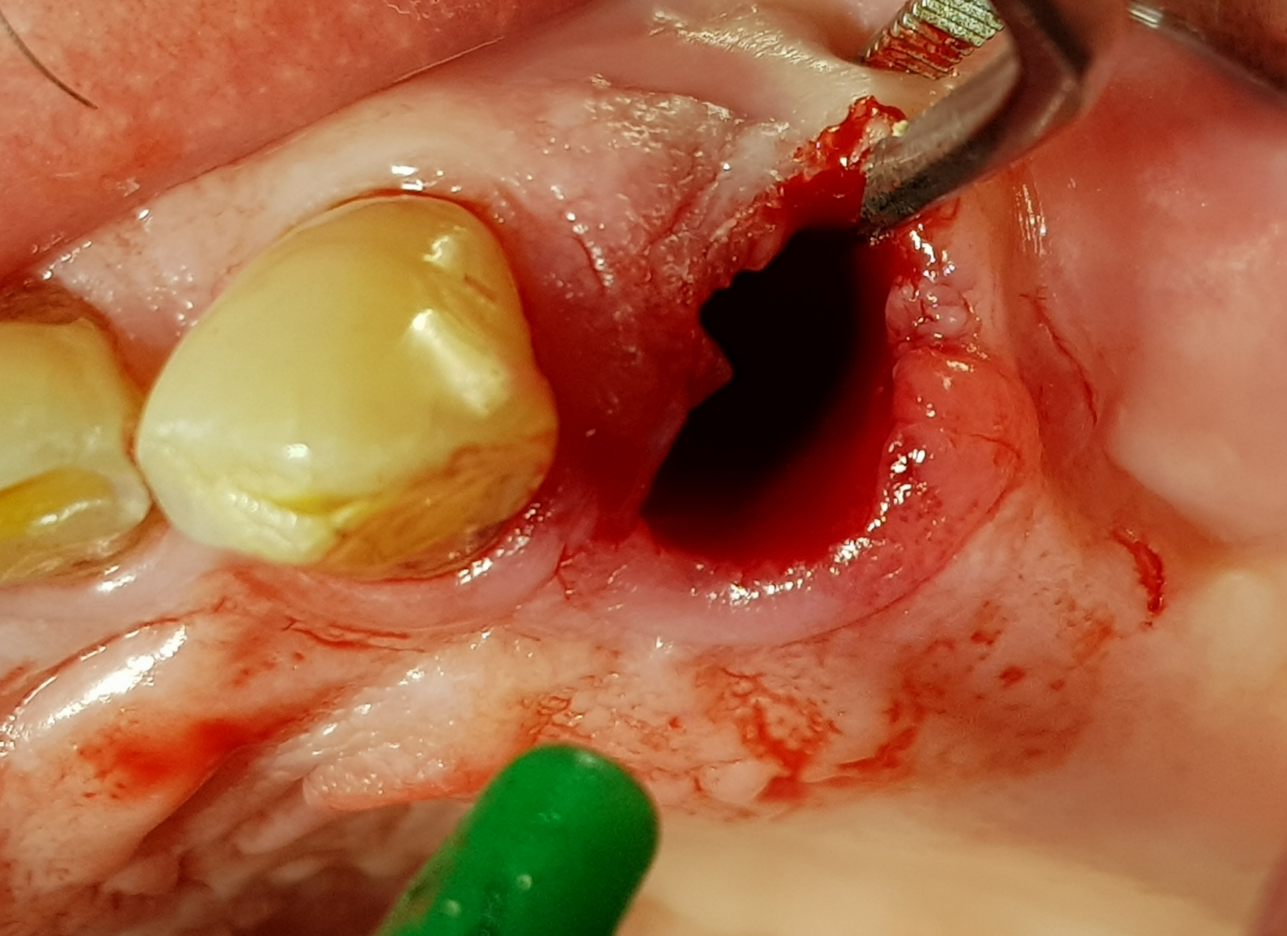
Extraction site following tooth extraction and curettage of the socket.

**Figure 2:**
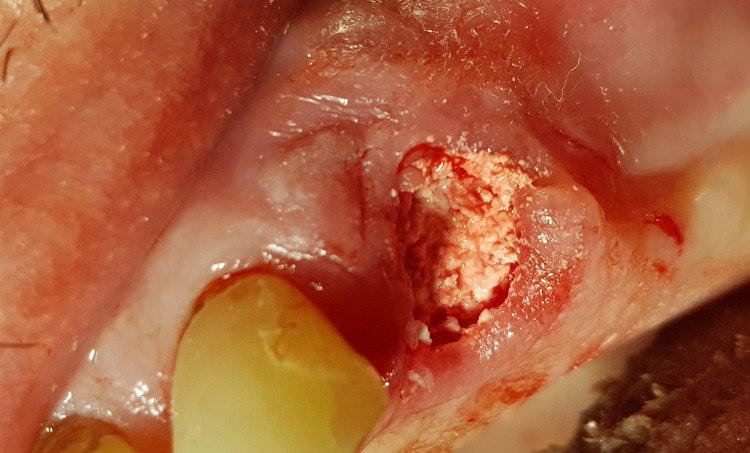
Extraction socket following placement of 3D Bond.

**Figure 3:**
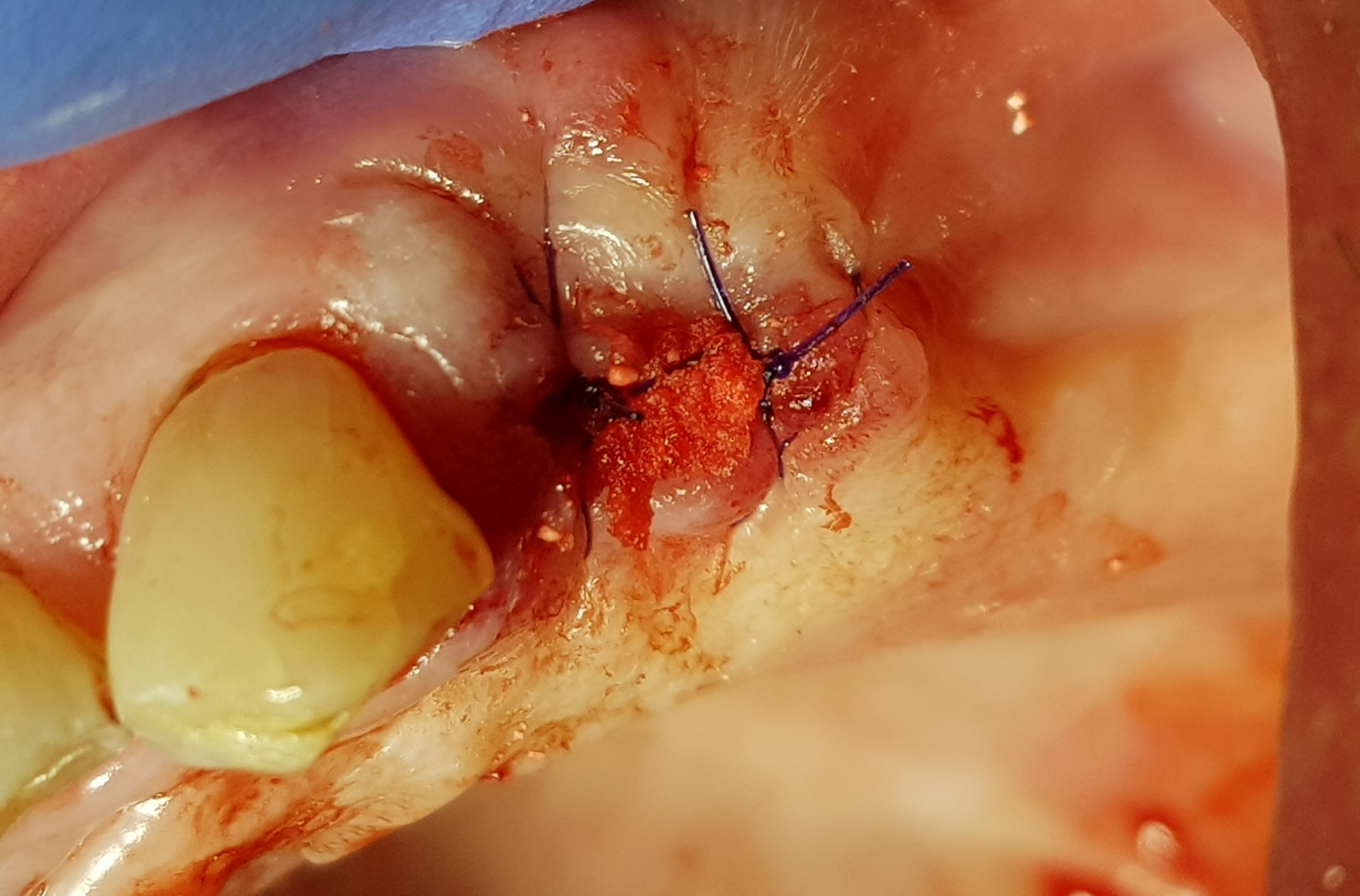
Suture placed across the socket with some graft exposure noted.

**Figure 4:**
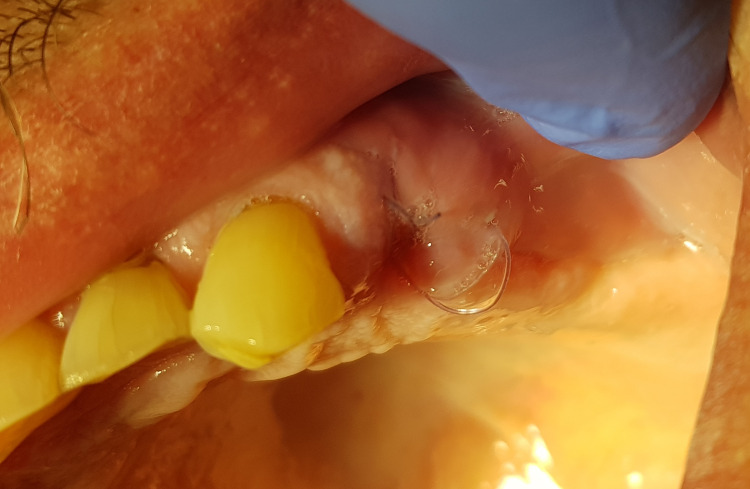
The extraction site at seven days post-extraction demonstrating an absence of gingival inflammation and bleeding.

All tooth extractions were performed under local anesthesia (4% articaine). Erupted teeth, impacted teeth, and roots were removed atraumatically. Based on previous experience, one or two teeth or roots were removed during a single treatment session. After removing the inflamed tissue from the extraction socket (Figure 1), 3D Bond® graft material was placed into the extraction socket and well compacted using dry sterile gauze and compression on the crest (Figure 2). The graft was covered only by a single compressed standard collagen sponge, and single stitches of sterile monofilament strands 4-0 or 5-0 were used to protect the xenograft material. Then, single sutures utilizing polydioxanone (resorbable monofilament) were placed across the crest (Figure 3). A resulting exposure of the graft material is allowed as the material sets hard, and soft tissue will migrate across the exposed material during initial healing without loss of graft material in the interim (Figure 4).

A total of 24 extractions were performed in the control group with 18 erupted teeth, three impacted teeth, and three teeth with pulpal necrosis. The same surgical procedure was performed in the control group, but without the utilization of socket grafting with 3D Bond. Those control cases received placement of a standard collagen dressing applied to the extraction socket (Cutanplast Dental, Mascia Brunelli SpA, Milano, Italy). Moreover, the standard sutures of polyglycolic acid were used in each case of the control. In the control group, all patients were administered amoxicillin with clavulanic acid in “two shots," with the first dose of 2.0 g per os administered two hours prior to surgery and a second dose of 1.0 g administered up to six hours after. Patients who had impacted teeth received amoxicillin for five days following surgery.

A total of 24 extractions were carried out in the study group, with 15 erupted teeth, two impacted teeth, and seven teeth with pulpal necrosis. Antibiotics were not used in the study group, except for patients with artificial cardiac valves. Those received amoxicillin with clavulanic acid in “two shots.” In addition, patients who had impacted teeth received amoxicillin for five days following surgery.

Local bleeding from the sockets was evaluated in both groups with a three-point scale: (0)-no bleeding; I°-seepage or bleeding needing drops of mouthwash tranexamic acid solution for three to five days; and II°-bleeding requiring a larger volume of the above, local procurement and optionally general administration 5-10 mg vitamin K intramuscularly or per oral administration. Evaluation of local bleeding was carried out after 60 minutes and at two-, three-, five-, seven-, and 10-days following surgery. Discomfort and postoperative pain, as evidenced by patient questioning, were tested using the visual analog scale (VAS) (100 mm long by a 0 mm means no pain and 100 mm the severest possible pain). Post-operative wound healing was assessed visually using a two-point scale: 0-healing proceeds correctly, 1-wound exhibits inflammatory features. Sutures were removed at 10 days post-surgery. Detailed parameters and results in both groups were presented in Tables 1-4.

**Table 1:** General characteristics of the study group. CABG: coronary artery bypass graft surgery, INR: international normalized ratio.

Variable	Mean ± SD	Range	n (%)
Age (years)	65.5 ± 12.65	35-90	–
Weight (kg)	80.1 ± 12.05	58-106	–
INR	2.54 ± 0.43	1.80-3.34	–
Sex			
Female	–	–	9 (45%)
Male	–	–	11 (55%)
Type of dental extraction			
Root extractions	–	–	18 (90%)
Impacted teeth	–	–	3 (15%)
Teeth with gangrene of the pulp	–	–	3 (15%)
Anticoagulant therapy daily dose of anticoagulant (mg)			
Acenocoumarol	4.9 ± 1.76	4-8	9 (45%)
Warfarin	6.8 ± 2.52	5-10	11 (55%)
Cardiological conditions			
Atrial fibrillation	–	–	9 (45%)
Mechanical aortic valve	–	–	3 (15%)
Mechanical mitral valve	–	–	2 (10%)
Mechanical mitral and aortic valve	–	–	2 (10%)
CABG	–	–	2 (10%)
Volume of graft used per procedure (cc)	1.0 ± 0.5	0.5-1.0	–

**Table 2:** Detailed characteristics of the study group. CABG: coronary artery bypass graft. Note: In total, 24 teeth were extracted using 17 cc of local hemostatic material. Postoperative bleeding occurred in one patient (5%).

Patient	Sex (F/M)	Age (years)	Body weight (kg)	Type of chronic cardiac disease 1-atrial fibrillation, 2-pulmonary embolism, 3-artificial valve, 4-CABG	Type of oral anticoagulant: 1-warfarin, 2-acenocumarol	Daily dose of oral anticoagulant (mg)	INR value	Extracted teeth and their condition: tooth (T), impacted tooth (IT), root (R)	Hemostatic material: 3D Bond (cc)	Postoperative bleeding (0 = no, 1 = yes (day after surgery))
1	F	51	65	3	2	4	3.05	48 (IT)	1	0
2	M	90	69	1	1	5	2.61	46 (R)	1	0
3	F	52	70	3	2	4	2.97	38 (IT)	1	0
4	M	64	92	1	1	7.5	2.95	24 (R)	0.5	0
5	F	35	58	2	1	5	2.74	48 (IT)	1	0
6	F	53	71	1	2	4	2.12	43 (R), 44 (R)	1	0
7	M	76	82	1	2	4	1.80	14 (R)	0.5	0
8	F	55	79	2	1	10	3.07	34 (T)	0.5	0
9	M	77	87	1	2	4	1.81	32 (R)	0.5	0
10	M	67	94	3	2	4	2.60	17 (R)	1	0
11	M	68	99	3	2	4	2.60	48 (R)	0.5	0
12	F	61	82	1	1	10	2.05	13 (R)	1	0
13	F	73	64	1	2	4	2.51	15 (R)	1	0
14	M	75	80	1	1	5	2.69	37 (T)	1	1 (3 day)
15	M	79	82	3	1	5	2.44	32 (R), 31(R)	1	0
16	M	76	86	1	1	10	2.01	12 (R), 11(R)	1	0
17	M	68	77	1	1	5	2.61	46 (R)	1	0
18	F	70	78	4	1	5	2.72	11 (R), 21(R)	1	0
19	F	65	106	3	1	5	2.20	18 (T)	0.5	0
20	M	55	82	3	2	4	3.34	47 (R)	1	0

**Table 3:** General characteristics of the control group. CABG: coronary artery bypass graft surgery, INR: international normalized ratio.

Variable	Mean ± SD	Range (min-max)	n (%)
Age (years)	64.25 ± 9.11	49-87	–
Body weight (kg)	77.50 ± 13.59	54-110	–
INR (after discontinuation)	1.25 ± 0.17	0.97-1.51	–
Sex			
Female	–	–	11 (55%)
Male	–	–	9 (45%)
Type of dental extraction			
Root extraction	–	–	12 (60%)
Impacted tooth	–	–	2 (10%)
Tooth with gangrene of the pulp	–	–	6 (30%)
Anticoagulant therapy	–	–	–
Warfarin	–	–	9 (45%)
Acenocoumarol	–	–	11 (55%)
Enoxaparin (mg/day)	62.00 ± 15.76	40-80	20 (100%)
Cardiological conditions	–	–	–
Artificial cardiac valve	–	–	4 (20%)
CABG	–	–	4 (20%)
Pulmonary embolism	–	–	4 (20%)
Atrial fibrillation	–	–	8 (40%)

**Table 4:** Characteristics of the control group. CABG: coronary artery bypass graft, LMWH: low-molecular-weight heparin. Note: In total, 24 teeth were extracted using 82 collagen sponges (Cutanplast Dental, Mascia Brunelli SpA, Milano, Italy). Postoperative bleeding occurred in four patients (20%).

Patient	Sex (F/M)	Age (years)	Body weight (kg)	Type of chronic cardiac disease: 1-atrial fibrillation, 2-pulmonary embolism, 3-artificial valve, 4-CABG	Type of oral anticoagulant: 1-warfarin, 2-acenocumarol/LMWH	Daily dose of oral anticoagulant (mg)	Bridging therapy: Enoxaparin (mg/day)	INR value 10 days after discontinuation and one day before surgery	Extracted teeth and their condition: tooth (T), impacted tooth (IT), root (R)	Hemostatic material: collagen sponge 10×10×10 mm (n)	Postoperative bleeding (0 = no, 1 = yes (day after surgery))
1	F	64	54	3	2	4	60	1.05	38 (IT)	4	0
2	M	52	79	3	1	5	80	0.97	38 (T)	3	0
3	M	87	71	4	1	5	40	1.12	45 (R)	2	0
4	M	66	89	1	2	4	80	1.15	24 (R)	3	0
5	F	59	68	2	1	5	60	1.14	18 (T)	3	0
6	M	63	72	1	2	4	60	1.12	46 (R)	6	1 (3 day)
7	M	76	100	1	2	8	80	1.49	16 (R)	4	1 (3 day)
8	M	55	110	2	1	10	80	1.51	34 (R)	4	1 (3 day)
9	F	71	83	1	2	4	60	1.36	41 (R)	2	0
10	F	67	69	4	2	4	40	1.32	14 (R)	3	0
11	M	65	84	3	2	4	60	1.38	48 (R)	4	0
12	F	62	70	1	1	10	60	1.42	33 (T)	4	0
13	F	68	92	2	1	10	80	1.30	34 (R), 35 (R)	4	0
14	F	59	75	1	2	8	60	1.24	26 (T)	4	0
15	M	73	81	4	1	5	80	1.12	47 (T)	4	1 (3 day)
16	F	60	68	3	1	5	40	1.08	15 (R), 16 (R)	6	0
17	F	73	74	1	2	8	60	1.44	23 (IT)	8	0
18	M	64	86	2	1	5	80	1.50	36 (T)	4	0
19	F	52	67	1	2	4	40	1.12	33 (R), 34 (R)	6	0
20	F	49	58	4	2	4	40	1.21	48 (R)	4	0

## Results

For quantitative variables, basic measures of location and variability were calculated. The significance of differences between groups was tested after verifying the assumptions of conformity of the empirical distributions with the normal distribution (Shapiro-Wilk test), using the Student’s t-test. In the absence of variance homogeneity, the t-test with Cochran-Cox adjustment was employed. The Mann-Whitney U test (Z or U) was utilized in other cases. To assess the significance of differences between percentages in subgroups, a test for differences between two structural indices was used. Relationships between characteristics were tested using the chi-squared test or chi-squared with Fisher's adjustment. Depending on the details of the hypotheses, a one-tailed or two-tailed critical area was tested. The study used a significance level of α = 0.05. The results are presented in Tables 5-12, including method, results, comparison between the 3D Bond study group and the collagen sponge, control group (N = 40), and the characteristics of both groups with and without bleeding after surgery.

Forty patients were included in the analysis: 20 in the 3D Bond study group and 20 in the control group. No significant differences were observed between groups in terms of age (65.50 ± 12.65 vs 64.25 ± 9.11 years; p = 0.722), body weight (80.15 ± 12.05 vs 77.50 ± 13.59 kg; p = 0.518), or sex distribution (p = 0.527) (Table 5). Similarly, no significant differences were found between groups regarding underlying cardiovascular conditions or anticoagulant dose (Table 6).

**Table 5:** General characteristics of patients undergoing surgery: comparison between 3D Bond study group and collagen sponge control group (N = 40). Note: Continuous variables are presented as mean ± SD and were compared using Student's t-test. Categorical variables are presented as n (%) and were compared using the Chi-square test.

Variable	Study (n = 20)	Control (n = 20)	Test statistic	p-value
Age (years)	65.50 ± 12.65	64.25 ± 9.11	t = 0.36	0.722
Body weight (kg)	80.15 ± 12.05	77.50 ± 13.59	t = 0.65	0.518
Sex			χ^2 ^= 0.40	0.527
Female	9 (45%)	11 (55%)		
Male	11 (55%)	9 (45%)		

**Table 6:** Cardiological treatment of patients undergoing surgery: comparison between 3D Bond study group and collagen sponge control group (N=40). Note: Categorical variables are presented as n (%) and were compared using the Z test for two independent proportions or Fisher's exact test, as appropriate for sparse data. Anticoagulant doses are presented as median (IQR) and were compared using the Mann–Whitney U test. CABG: coronary artery bypass graft surgery.

Variable	Study (n = 20)	Control (n = 20)	Test statistic	p-value
Atrial fibrillation	10 (50%)	12 (60%)	Z = -0.64	0.525
Pulmonary embolism	2 (10%)	4 (20%)	Fisher exact	0.376
Mechanical aortic valve	5 (25%)	4 (20%)	Z = 0.37	0.705
Mechanical mitral valve	4 (20%)	2 (10%)	Fisher exact	0.376
CABG	2 (10%)	4 (20%)	Fisher exact	0.376
Two cardiological diseases/interventions	3 (15%)	6 (30%)	Z = -1.14	0.256
Anticoagulant dose (mg/day)	5.0 (4.0–8.0)	5.0 (4.0–8.0)	Z = -0.27 (Mann–Whitney)	0.787

As expected, significant differences in coagulation parameters were observed between groups (Table 7). Patients in the study group (without anticoagulant discontinuation) presented significantly higher INR values compared with controls (2.54 ± 0.43 vs 1.25 ± 0.17; p < 0.001), with a very large effect size (η² = 0.86).

**Table 7:** Characteristics of groups with and without bleeding after surgery (N = 40). Note: Data are presented as mean ± SD or number (percentage of group), as appropriate. The t-test was used for continuous variables and Fisher's exact test for categorical variables. Effect size was expressed as partial eta-squared (η²) for t-tests. Odds ratio (OR) was estimated with Haldane-Anscombe correction due to a zero cell.

Variable	No bleeding (n=35)	Bleeding (n=5)	Test statistic	p-value	Effect size
Age (years)	64.37 ± 11.15	68.4 ± 9.11	t = -0.77	0.447	η^2^ = 0.02
Body weight (kg)	77.43 ± 11.88	88.6 ± 15.8	t = -1.89	0.066	η^2^ = 0.09
Sex			Fisher's exact test	0.047	OR = 14.55 (95% CI: 0.75-283.39)
Female	20 (57.14%)	0 (0%)			
Male	15 (42.86%)	5 (100%)			

Prothrombin time (PT) was significantly shorter in the study group (median 34.15 (30.70-50.95) vs 99.26 (88.65-120.95); p < 0.001; r = 0.87). Activated partial thromboplastin time (APTT) was also significantly higher in the study group (54.08 ± 9.79 vs 38.64 ± 5.13; p < 0.001; η² = 0.58). These results confirm substantial differences in coagulation status between groups prior to surgery.

No significant differences were observed between groups regarding the type of dental intervention (p = 0.508). Although 24 teeth were removed in each group, the distribution of removed tooth types differed significantly between groups (p = 0.043, Cramér's V = 0.41) (Table 8). Specifically, incisors were more frequently extracted in the study group, whereas bicuspids were more common in the control group. However, these differences were not associated with postoperative bleeding.

**Table 8:** Details of dental surgery: comparison between 3D Bond study group and collagen sponge control group (N = 40). Note: Data are presented as number (percentage of group) or median (IQR), as appropriate. The Chi-square test was applied for categorical variables. The distribution of removed tooth types was analyzed per extracted tooth using the Pearson Chi-square test. Effect size for the 2×4 table was expressed as Cramér’s V. Hemostatic material was determined by group allocation and therefore was not statistically compared.

Variable	Study	Control	Test statistic	p-value	Effect size
Type of dental intervention (per patient)	(n = 20)	(n = 20)	χ² = 1.35	0.508	-
Root extraction	14 (70%)	12 (60%)			
Extraction due to pulp gangrene	3 (15%)	6 (30%)			
Impacted tooth extraction	3 (15%)	2 (10%)			
Type of removed tooth (per tooth)	(n = 24)	(n = 24)	χ² = 8.13	0.043	V = 0.41
Incisors	7 (29%)	1 (4%)			
Cuspid	3 (13%)	4 (17%)			
Bicuspid	4 (17%)	11 (46%)			
Molar	10 (42%)	8 (33%)			
Hemostatic material used			-	-	-
3D Bond (cm³)	1.0 (0.5-1.0)	-			
Collagen sponge (10×10×10 mm)	-	4.0 (3.0-4.0)			

In the study group, postoperative bleeding occurred in one patient (5%), whereas in the control group, bleeding was observed in four patients (20%). Overall, five bleeding events (12.5%) were recorded in the entire cohort. No significant association was found between the type of removed tooth and postoperative bleeding (per tooth analysis, n = 48; χ² = 3.23, p = 0.358).

In this research, bleeding occurred exclusively in male patients. The association between male sex and postoperative bleeding reached statistical significance (p = 0.047), with an odds ratio of 14.55 (95% CI: 0.75-283.39). However, the confidence interval was wide due to the small number of bleeding events (Table 9). No significant differences were found between patients with and without bleeding in terms of age (p = 0.447; η² = 0.02) or body weight (p = 0.066; η² = 0.09).

**Table 9:** Preparation of groups for dental surgery: comparison between 3D Bond study group and collagen sponge control group (N = 40). Note: Data are presented as mean ± SD or median (IQR), as appropriate. Student's t-test with Welch's correction and the Mann-Whitney U test were used to estimate differences between groups. Effect size was expressed as partial eta-squared (η²) for t-tests and as r (Z/√N) for the Mann-Whitney test. INR: international normalized ratio, APTT: activated partial thromboplastin time, PT: prothrombin time.

Variable	Study (n = 20)	Control (n = 20)	Test statistic	p-value	Effect size
INR	2.54 ± 0.43	1.25 ± 0.17	t = 12.48	<0.001	η^2^ = 0.86
PT (s)	34.15 (30.70–50.95)	99.26 (88.65–120.95)	Z = -5.50	<0.001	r = 0.87
APTT (s)	54.08 ± 9.79	38.64 ± 5.13	t = 6.25	<0.001	η^2^ = 0.58

No significant differences were found between patients with and without bleeding in terms of cardiological treatment (p = 0.443), type of anticoagulant (p = 0.658), or anticoagulant dose (p = 0.499) (Table 10).

**Table 10:** Cardiological treatment in patients with and without post-surgical bleeding (N = 40). Note: Data are presented as numbers (percentage of group) or median (IQR), as appropriate. Fisher's exact test was used for categorical variables. The Mann–Whitney U test was applied for the anticoagulant dose.

Variable	No bleeding (n = 35)	Bleeding (n = 5)	Test statistic	p-value
Two cardiological diseases/interventions	8 (22.9%)	1 (20.0%)	Fisher's exact	0.443
Types of anticoagulants			χ^2^ = 0.84	0.658
Syncumar	4 (11.4%)	1 (20.0%)		
Acenocoumarol	14 (40.0%)	1 (20.0%)		
Warfarin	17 (48.6%)	3 (60.0%)		
Anticoagulant dose (mg/day)	5.0 (4.0–8.0)	5.0 (5.0–8.0)	U = 70.0	0.499

Within the control group (N = 20), no significant associations were found between bleeding and INR classification (p > 0.999), PT classification (p = 0.549), or APTT classification (p > 0.999) (Table 11). Patients with bleeding tended to receive higher doses of enoxaparin (median 80.0 vs 60.0 mg/day), although this difference did not reach statistical significance (p = 0.080; r = 0.27).

**Table 11:** Comparative preparation for surgery in patients with and without post-surgical bleeding (n = 20). Note: Data are presented as median (IQR) or number (percentage of group), as appropriate. The Mann–Whitney U test was applied for enoxaparin dose; effect size was expressed as r (Z/√N). Fisher's exact test was used for categorical variables. INR: international normalized ratio, APTT: activated partial thromboplastin time, PT: prothrombin time.

Variable	No bleeding (n = 16)	Bleeding (n = 4)	Test statistic	p-value	Effect size
Enoxaparin (mg/day)	60.0 (40.0-70.0)	80.0 (70.0-80.0)	U = 13.5	0.080	r = 0.27
INR			Fisher's exact test	>0.999	
Standard	7 (43.75%)	2 (50%)			
Above	9 (56.25%)	2 (50%)			
PT			Fisher's exact test	0.549	
Standard	12 (75%)	2 (50%)			
Above	4 (25%)	2 (50%)			
APTT			Fisher's exact test	>0.999	
Standard	10 (62.5%)	2 (50%)			
Above	6 (37.5%)	2 (50%)			

No significant association was found between postoperative bleeding and the overall type of dental intervention (p = 0.469). Similarly, bleeding was not associated with jaw location, number of extracted teeth, or type of removed tooth (Table 12). Furthermore, the difference in bleeding rates between the study and control groups did not reach statistical significance (p = 0.342).

**Table 12:** Characteristics of dental surgery in patients with and without post-surgical bleeding (N = 40). Note: Data are presented as number (percentage of group). The Pearson chi-square test was applied for multi-category comparisons (type of dental intervention and type of removed tooth). Fisher's exact test was used for binary categorical variables with small expected cell counts.

Variable	No bleeding (n = 35)	Bleeding (n = 5)	Test statistic	p-value
Type of dental intervention (per patient)			χ^2^ = 1.51	0.469
Root extractions	23 (65.7%)	3 (60.0%)		
Extraction due to pulp gangrene	7 (20.0%)	2 (40.0%)		
Impacted teeth	5 (14.3%)	0 (0%)		
Lower jaw	20 (57.1%)	4 (80.0%)	Fisher's exact test	0.813
Two removed teeth (per patient)	7 (20.0%)	1 (20.0%)	Fisher's exact test	>0.999
Group (per patient)			Fisher's exact test	0.342
Study	19 (54.3%)	1 (20.0%)		
Control	16 (45.7%)	4 (80.0%)		
Type of removed tooth (per tooth)	n = 43	n = 5	χ^2^ = 3.23	0.358
Incisor	8 (19%)	0 (0%)		
Cuspid	7 (16%)	0 (0%)		
Bicuspid	12 (28%)	3 (60%)		
Molar	16 (37%)	2 (40%)		

In the study group of 19 patients, 95% (11 males and eight females) reported no bleeding, despite disturbed hemostatic parameters confirmed by laboratory measurements. In one case, 5% (one male), a postoperative bleeding (I°) occurred on day three, which was controlled in an outpatient setting with tranexamic acid used intraorally, three times daily for one day. In the control group, 16 patients (80%; 11 females and five males) had no bleeding. Yet, in four cases, 15% (four males), postoperative bleeding (II°) occurred on day three. However, the INR indicator was normal in two cases and higher in two cases than the reference values. Every case of bleeding in the control group was controlled in an outpatient setting. The wounds were reoperated on with sutures and collagen dressing being changed, and tranexamic acid was used intraorally three times daily for a period of three days. The patients were instructed to rinse their mouths for three minutes and repeat after a few minutes over the next few hours.

In the study group, VAS values for post-operative discomfort and pain ranged from 20 to 45 mm (mean 34), whereas in the control group, patients' values ranged from 35 to 65 mm (mean 46).

Moreover, at seven days post-op in the control group, impaired wound healing was observed; in four cases, 20% (four males), bleeding was observed. In order to improve wound healing, an antiseptic rinse (chlorhexidine) and a local surgical dressing (Alveogyl Septodont, Saint-Maur-des-Fossés, France) were utilized.

## Discussion

It is accepted that Warfarin discontinuation is associated with poor clinical outcomes in surgically treated patients who have been treated chronically with dihydroxycoumarin derivatives [7,8]. In addition, maintaining the anticoagulant therapy together with the topical application of some hemostatic substance may reduce the treatment time and cost of oral surgery [9-12].

The study presented here demonstrated that using 3D Bond® regenerative cement without warfarin or acenocoumarol discontinuation in the patients treated with planned oral surgery is safe and effective in this cohort of patients. Moreover, it potentially protected the extraction sockets from bone atrophy during site healing, but this was not the main topic of this study.

Based on the author’s experience in this kind or similar study topic, the graft keeps the stability of the shape in the ridge and mechanically closes the micro-vessels in the cancellous bone around and inside the extraction socket. In the past, a few groups of patients chronically using dihydroxycoumarin derivatives were examined by the authors. In one multicenter study, 560 tooth extractions were performed in a group of 293 patients. All of those patients were chronically taking acenocumarol or warfarin because of cardiovascular diseases. The authors used, among other things, collagen and a bovine bone formulation (Tablet®, Tecnoss-Dental, Torino, Italy) and compressive gauze with tranexamic acid up to 60 minutes post-op. The overall bleeding rate was 6.8%. However, those patients who had more than two extractions were at significantly higher risk of localized bleeding. With regard to bone regeneration, only the integration of xenografts with human host bone was observed radiographically [13].

Also, in the author's study in 2021, comparable results were obtained in supporting hemostasis. All study patients were referred for urgent oral surgery. PARASORB Cone® collagen (iRES® SAGL, Switzerland) was used in 30 patients after 66 extractions, compared with the control group (50 tooth extractions), in which bridging therapy was used, with the anticoagulant drug replaced by low-molecular-weight heparin administered subcutaneously. In both groups, three local bleedings occurred on the second and third day. Although the authors believe that collagen is highly effective, the properties of the 3D Bond hardening bone cement are more advanced and enable greater predictability of the procedure and wound healing [13]. Additionally, the authors advanced the thesis that it could stimulate coagulation factors as it contains calcium ions that can potentially induce the coagulation pathway. But this requires expanding the panel of in vitro tests in that direction.

Similarly to the author’s study, Bacci also reported no significant differences in the incidence of bleeding after extraction in 451 patients taking Warfarin (INR 1.8-4.0) compared with a group of 449 healthy subjects that underwent identical surgical procedures [10]. In patients who continued anticoagulant therapy, topical hemostatic, collagen sponges, and closure with sutures, fibrin, and compression with sterile gauze soaked with tranexamic acid were utilized. Postoperative bleeding was experienced in both the study group (n = 7) and the control group (n = 4). None of the complications required hospitalization or blood transfusions. They also considered it safe for the patients to continue warfarin treatment if topical hemostatic measures were taken to reduce discomfort to the patients. Keiani Motlagh reported the same experience in patients and recommended an oral rinse with a 5% acid solution of tranexamic during and after dental surgery [12]. No local bleeding was reported.

Study limitations

This study has several limitations that should be considered when interpreting the findings. The sample size was relatively small and derived from a single clinical setting, which may limit generalizability. The control group was retrospective in nature and treated during a different time period, introducing potential selection and procedural variability. Additionally, although clinical bleeding outcomes were assessed using standardized criteria, more objective quantitative measurements of blood loss were not performed. The study was not designed to evaluate long-term regenerative outcomes, and radiographic or histomorphometric analyses were not included. Future prospective, randomized, multicenter investigations with larger cohorts and standardized bleeding assessment protocols are warranted to further validate these findings.

## Conclusions

Within the limitations of this clinical study, the use of biphasic calcium sulfate cement (3D Bond®) provided effective local hemostasis following oral surgical procedures in patients maintained on vitamin K antagonist therapy. The findings support the clinical feasibility of performing extractions without interruption of systemic anticoagulation when appropriate local hemostatic protocols are implemented.

The ability to maintain anticoagulant therapy while achieving predictable bleeding control has important implications for patient safety and workflow efficiency. In addition to hemostatic control, clinical observations suggest potential regenerative benefits in extraction sockets, supporting its dual role in both surgical management and post-extraction site preservation.
